# Atlantic overturning: new observations and challenges

**DOI:** 10.1098/rsta.2022.0196

**Published:** 2023-12-11

**Authors:** Meric A. Srokosz, N. Penny Holliday, Harry L. Bryden

**Affiliations:** ^1^ National Oceanography Centre, Southampton SO14 3ZH, UK; ^2^ School of Ocean and Earth Science, University of Southampton, Empress Dock SO14 3ZH, UK

**Keywords:** Atlantic, overturning circulation, observations, AMOC, RAPID, OSNAP

## Abstract

This paper provides an introduction to the special issue of the Philosophical Transactions of the Royal Society of London of papers from the 2022 Royal Society meeting on ‘Atlantic overturning: new observations and challenges'. It provides the background and rationale for the meeting, briefly summarizes prior progress on observing the Atlantic overturning circulation and draws out the new challenges that papers presented at the meeting raise, so pointing the way forward for future research.

This article is part of a discussion meeting issue 'Atlantic overturning: new observations and challenges'.

## 1. Introduction

**Figure 1.**
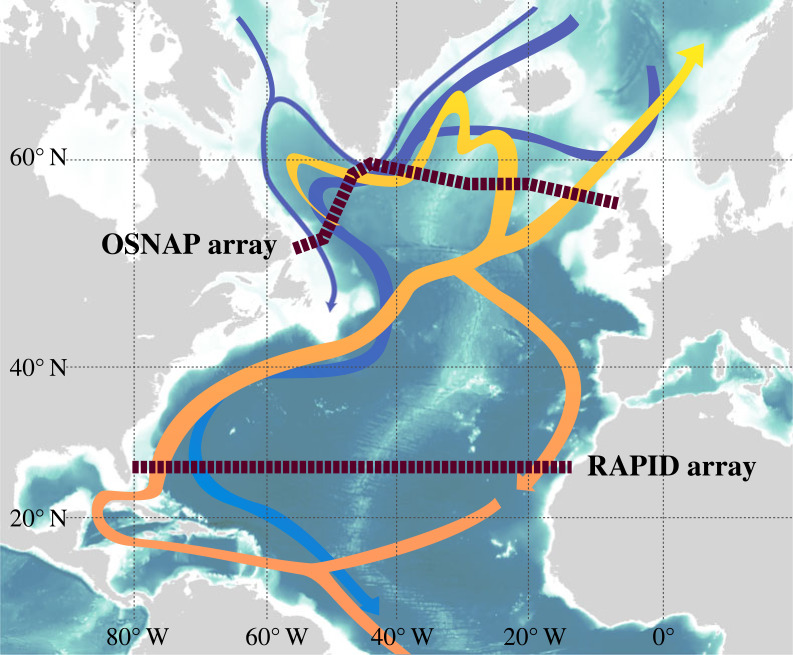
Highly simplified schematic of the overturning in the North Atlantic showing the location of the OSNAP and RAPID observing arrays. Orange and yellow colours depict the warm surface waters flowing north, while blue colours show the deep return flows.

In 2003, the Royal Society hosted a meeting entitled ‘Abrupt climate change: evidence, mechanisms and implications', the proceedings of which were published in the *Philosophical Transactions of the Royal Society of London A* (**361**, 1827–2078). The Atlantic thermohaline circulation (THC) was thought to be responsible for some of the abrupt climate changes that had been found in paleo data; abrupt being defined as a change on time scales of the order of a decade or less rather than the 100-year time scale associated with climate predictions/projections, such as those generally considered by the Intergovernmental Panel on Climate Change (IPCC). The picture of the THC that had been put forward by Broecker ([1]; see also [2]), based on paleo data, was of a large-scale ‘conveyor belt’ circulation driven by changes in heat (thermo) and salt (haline; or equivalently freshwater) content. In the Atlantic, the circulation brought warm water north, giving up heat to the atmosphere, cooling and sinking as dense water at high northern latitudes and returning to the south as cold water at depth (figure 1). With global warming leading to more rainfall at high latitudes together with meltwater from Greenland, and warming of the ocean, it was thought that the surface waters would become less dense potentially preventing sinking and formation of deep return flow. In this way, it was believed that the conveyor could be disrupted, causing it to slow down or even stop, with serious climatic impacts [3,4]. Given the potential climatic impacts of such changes in the THC, the question of interest at the time was: How is the Atlantic circulation responding to climate change?

The Atlantic circulation consists of both a wind-driven and a thermohaline component and while it is possible to conceptually differentiate between the two it is difficult practically to separate the two using observations. Therefore, it is more helpful to think about the meridional overturning circulation (MOC), encompassing the effects of both the wind-driven and thermohaline contributions to the overturning flow. This is an aspect of the circulation that can be measured directly and more easily diagnosed from model output [5]. At the time of the 2003 meeting, no continuous observations of the Atlantic MOC (AMOC) existed and only ‘snapshots' of the circulation had been obtained from infrequent trans basin hydrographic cruises [6]. At the 2003 meeting plans were presented for a new programme, the Rapid Climate Change programme (*aka* RAPID; [7]), which would seek to make continuous measurements of the AMOC in the North Atlantic. This monitoring would be a joint venture between the Natural Environment Research Council (NERC) in the UK and the National Science Foundation (NSF) and National Oceanographic and Atmospheric Administration (NOAA) in the USA. The monitoring of the AMOC started in 2004 under the aegis of the RAPID programme and led to many new observations, insights and challenges with respect to the Atlantic overturning circulation.

The purpose of the present meeting was to explore what had been learnt since the 2003 meeting and to assess what gaps in understanding and challenges remain with regard to AMOC. This introductory paper provides some background to the meeting and a brief overview of the topics covered during the meeting, ending with a look at the way forward into the future.

As well as the papers presented at this meeting, there are also a number of review papers that the reader can consult for more detailed background on what follows. These include: Buckley & Marshall [8]; Srokosz *et al*. [9] and the papers listed therein, which introduces the American Geophysical Union (AGU) journals' special issue on AMOC observational and modelling advances; and papers by Bryden, Weijer *et al*. and Jackson *et al*. [10–12].

## 2. Atlantic overturning

The AMOC, which can be characterized as the northward flow of warmer saltier water in the upper Atlantic and the southward flow of the colder fresher North Atlantic Deep Water (NADW) in the deep Atlantic, transports a substantial amount of heat northward across the equator in the Atlantic. In this respect, it differs radically from the Indian and Pacific Oceans, where the heat transport is away from the equator and towards poles [13,14]. It has long been known from Stommel's [15] classic paper that the THC, and so the AMOC too, are potentially unstable and could ‘flip’ modes between ‘on' and ‘off'. Whether this is in fact possible for the real-world circulation is unclear (see [16], for a recent review of the stability of the AMOC), but if it were to happen the climate impacts would be substantial.

The climatic impacts of changes in the AMOC are many, either directly or via its link to the Atlantic Multidecadal Variability (AMV; [17,18]). The significant impacts include: changes in sea level; the ability of the North Atlantic to act as a carbon sink; Intertropical Convergence Zone shifts; Sahel and Indian monsoons; frequency and strength of Atlantic hurricanes; El Niño–Southern Oscillation; Pacific Decadal Variability; North Atlantic Oscillation and storm tracks; climate over Europe, North America and Asia; Arctic sea ice and surface air temperature; and global surface temperature changes. In addition, paleoclimatic evidence indicates that a link between multi-decadal AMOC variability and AMV and many associated climate impacts may also have existed in the preindustrial era [18]. Many, if not all, of these impacts have important socio-economic implications and therefore underline the importance of understanding the past, present and future behaviour of the AMOC.

As noted earlier, the IPCC's 2001 report [3] raised concerns about the future behaviour of the AMOC as the coupled climate models at that time were predicting that it would decline. The IPCC 2019 special report on the ocean and cryosphere [19] states that ‘Observations, both *in situ* (2004–2017) and based on sea surface temperature reconstructions, indicate that the AMOC has weakened relative to 1850–1900 (*medium confidence*)', and that ‘The AMOC is projected to weaken in the 21st century under all RCPs (*very likely*), although a collapse is very unlikely (*medium confidence*)'. The latest IPCC 2021 report [20] states that ‘While there is medium confidence that the Atlantic Meridional Overturning Circulation (AMOC) will not experience an abrupt collapse before 2100, if it were to occur, it would very likely cause abrupt shifts in regional weather patterns and water cycle' and ‘it is *very likely* that AMOC will decline over the 21st century'. The fact that the AMOC is projected to weaken under the latest IPCC scenarios and confidence in that projection has grown since 2001 (now considered *very likely*) means that going forward into the twenty-first century there will be climatic impacts as a result of that weakening. Suffice to say that knowing the present state of the AMOC, and its past behaviour, are both important if the impact of AMOC changes under global warming are to be predicted and taken into account in formulating governmental policy responses such as mitigation or adaptation.

## 3. New observations

**Figure 2.**
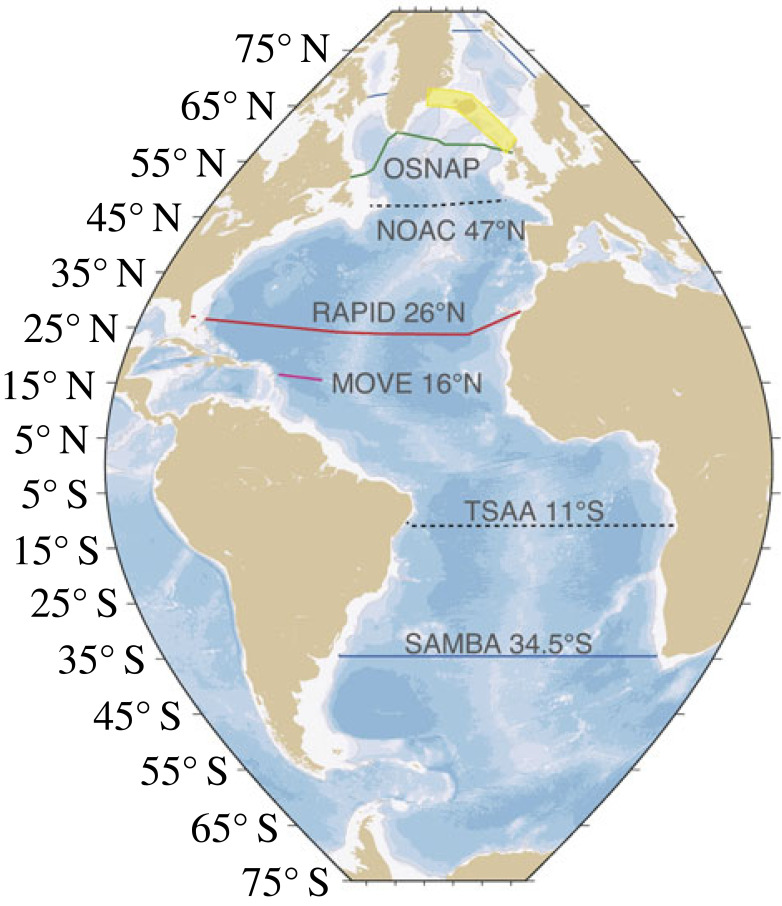
Locations of the various observing systems deployed in the Atlantic and in the Arctic gateway region (after fig. 3 of [25]; courtesy of Eleanor Frajka-Williams). The Atlantic arrays (OSNAP, NOAC 47° N, RAPID 26° N, MOVE 16° N, TSAA 11° S, SAMBA 34.5° S) are fully described by Frajka-Williams *et al*. [25]. For the yellow region, the Scotland-Iceland-Greenland observations, see Østerhus *et al*. [26], and for the Arctic gateway region further north see Tsubouchi *et al*. [27].

Since 2003 significant progress in observing the Atlantic overturning has been made. The RAPID programme's subtropical gyre 26° N observing system's success [21,22] gave impetus for the development of further observing systems to be deployed across the Atlantic at different locations. Further north in the Atlantic, in the subpolar gyre, the Overturning in the Subpolar North Atlantic Program (OSNAP) observing system was deployed (figure 1) and started making measurements of the overturning in 2014 [23]. In the South Atlantic at 34.5° S the South Atlantic MOC Basin-wide Array (SAMBA) observing system has been making measurements of the overturning since 2009 (but with a gap 2011–2013; [24]). Other observations and observing systems (figure 2) have been detailed and reviewed in an OceanObs'19 white paper by Frajka-Williams *et al*. [25].

Prior to the 2003 Royal Society meeting the accepted picture of the overturning circulation was of a large-scale ‘conveyor belt'-like flow as proposed by Broeker [1]. Whereas the picture that emerged, first from the RAPID observations at 26° N [21], and later from OSNAP in the subpolar gyre [23] and SAMBA in the South Atlantic at 34.5° S [24], was that of an overturning flow that is highly variable across a range of time scales from days to years. The results obtained from the AMOC observations over the years have fulfilled the promised ‘inevitable surprises' in the title of the NRC [4] report.

Among the ‘inevitable surprises' about the AMOC that the new observations have revealed are:

— the large AMOC variability on short time scales [21,23,24,28];
— the forcing and time scales of variability differ latitudinally, meaning that there is no single measure of AMOC strength that applies to the entire north to south Atlantic [25,29];
— the existence of significant subtropical and subpolar AMOC seasonal cycles [30,31];
— an unexpected approximately 30% (approx. 6 Sv) temporary downturn in 2009/2010 in the AMOC at 26° N [32];
— a faster decline than that predicted by climate models in the 2004–2009 period then a levelling out [33,34];
— the continuing reduced state of the AMOC at 26° N, reduced by approximately 12% (approx. 2 Sv) [35,36];
— at 26° N changes in LNADW transport (with origin in the Nordic Seas) at depths of 3000–5000 m rather than UNADW (waters from the Labrador Sea) at 1000–3000 m [10,35];
— OSNAP observations show that AMOC mean and variability in OSNAP East (Scotland to Greenland) dominates over that in OSNAP West (Greenland to Canada; [23,31]);
— density changes in the Irminger Sea are more important than those in the Labrador Sea for AMOC variability [37], whereas previously the high winter fluxes in the Labrador Sea were thought to be a key driver of AMOC variability;
— in addition to the southward deep flow in the Deep Western Boundary Current (DWBC, [38]) there are ‘interior pathways' that carry some of the deeper waters [39];
— determination of heat and freshwater transports in the subpolar and subtropical and S. Atlantic, and their strong relationship to the AMOC [28,31,40–42];
— the importance of the AMOC for nutrient and anthropogenic carbon transports and effects on carbon uptake in the North Atlantic [43,44];
— diapycnal mixing is as important as air–sea fluxes in the process of transforming warm saline water into the deep water masses of the AMOC southward flow, and therefore in setting the mean strength of the subpolar AMOC [45].

These discoveries from the new observations have challenged previous AMOC paradigms including the following: that the AMOC is driven by thermohaline changes and varies on time scales of years to millennia; that changes in convection in the Labrador Sea play a key role in the AMOC; and that the deep return flow is confined to the Deep Western Boundary Current (DWBC). It is now known that the AMOC is highly variable on all time scales, from days upwards, and that much of that shorter time-scale variability is wind forced, while thermohaline forcing is important for longer time scales. Some of the AMOC return flow is via interior pathways in both the North and South Atlantic, away from the western boundary, making the AMOC less meridionally coherent. These paradigm shifts and ‘inevitable surprises' have led to new challenges, to which we now turn.

## 4. New challenges

The papers presented at the meeting, and contributed to this special issue, update and review recent progress in observing and modelling the AMOC. Here we summarize some of the key new challenges, noted at the meeting and in the papers in this volume, regarding observing and modelling the AMOC that arise from what has been discovered to date.

One thing that emerges is that the *in situ* observational record is too short as yet to definitively answer some of the outstanding questions, and specifically the question of how representative the observations are of longer-term AMOC variability. This means that observations of the AMOC need to continue in some form or another, but it is currently unclear what that form should be [46]. Observations at some locations in the Atlantic are more challenging [47] than at others [48] due to the complexities of the flow and the topography. Extending the observational record back in time by the use of proxies presents its own challenges [49], especially as different paleo proxies provide information on different aspects of the AMOC [50]. A further challenge is how to combine the various observations and to standardize methodologies, to give a complete and consistent picture of the Atlantic overturning and address the question of meridional coherence. Part of the question about methodology choices is whether the AMOC metrics are best given in depth or density coordinate representation. Overturning in density coordinates is closely related to the overturning process of diapycnal water mass transformation, which is of particular importance in the subpolar AMOC [51].

As it is ocean heat transport (OHT) rather than the AMOC *per se* that is the climatically important quantity, the relationship between OHT and AMOC has been a special focus in studies to date [47,48]. However, there are indications from the RAPID measurements at 26° N that this relationship can change, and this raises the important question of how it might change under future global warming [48]. Similarly, given the importance of the AMOC for anthropogenic CO_2_ uptake, another climatically important quantity (Brown, meeting presentation), how will future change affect the CO_2_ uptake? A complex issue is that of changes in salinity and associated salinity (or equivalently freshwater) transport (de Jong, meeting presentation). For example, will the arrival of significantly fresher water in the subpolar North Atlantic [52,53] impact the AMOC or not? Furthermore, small measurement errors of 0.003 in salinity can lead to uncertainties in AMOC estimates of approximately 1 Sv at 26° N where the Atlantic is at its widest [54], so continuing highly accurate observations represents an ongoing calibration challenge.

It is known that changes to freshwater in the subpolar North Atlantic are happening [52,53] and model experiments have shown that increased freshwater input could disrupt the AMOC [55]. Therefore, an unknown aspect of the Atlantic overturning is how glacial melt from Greenland [47] and/or freshwater export from the Artic [56] might impact the AMOC and on what time scale. Furthermore, it is unknown on what time scales, and by which processes, changes in deep water formation at high latitudes might propagate south. For example, the question of the importance of advective versus wave processes remains open [57]. An understanding of the export of NADW from the subpolar region to the subtropics is important for characterizing the behaviour of the large-scale AMOC. Careful observations in the subtropical–subpolar gyre transition could shed new light on how signals of change in NADW propagate southwards [51,58].

Questions remain too about current understanding of the processes that impact the AMOC. For example, the degree to which surface buoyancy forcing versus wind forcing, either remotely or locally, affects the AMOC and on what time scales [47,48,59]. Furthermore, there is a clear distinction between deep convective mixing and sinking and the two may not necessarily occur in the same location. Therefore, more work is required to link the processes of air–sea interaction, water mass formation and sinking that determine the lower limb of the overturning [57]. For example, understanding the formation and export of Labrador Sea Water as a significant pathway for CO_2_ to enter the deep ocean is a key linkage to the AMOC [60].

Moving on to the issues associated with modelling the Atlantic overturning, it is acknowledged that the CMIP6 models (that is, the state-of-the-art coupled climate models—at the time of writing—used as the basis of the AR6 IPCC 2021 report) have clear limitations in their representation of the AMOC [49,61]. Furthermore, the IPCC focus has been on the future behaviour of the AMOC but perhaps of more concern is that the models tend to underestimate the strength of the OHT [48]. Given that the current IPCC models are deficient in some key processes, such as Greenland meltwater entering the North Atlantic, it is unclear whether they will be able to reproduce the sort of abrupt AMOC changes that are seen in paleo data.

It is known that models suffer from a variety of biases, especially in the Labrador Sea and subpolar North Atlantic, and some of these can be addressed by increasing the resolution of the ocean component to capture eddy processes, though at higher latitudes this becomes more challenging due to the decrease in Rossby radius [61]. Other biases like those due to the representation of deep overflows may be harder to deal with, and the biases may be model dependent. Thus, both higher resolution models and new model developments are required to better represent the AMOC. In terms of future projections, a way forward may be to use observational constraints to sub-select models and model ensemble members using history matching [49], as has recently been done in studying future Arctic sea ice decline [62].

With regard to paleo modelling, one way forward is to use data assimilation to make best use of the sparse paleo data that are available. This requires careful determination of uncertainties in both the paleo observations and the models, and an awareness of the fact that different paleo proxies constrain different aspects of the overturning. It remains unclear whether insights for paleo modelling will be helpful in determining the near-term (centennial) changes in the AMOC [50].

As noted earlier, some of the climatic impacts of AMOC changes are mediated via the AMV, so understanding the AMOC–AMV link is an important issue [63]. A particular challenge in modelling the AMV, and its associated impacts, is the large uncertainty in the magnitude of the simulated historical forcing, specifically the anthropogenic aerosol forcing.

## 5. The future

The papers presented at the meeting (see the list below) highlight the progress made in observing and understanding the Atlantic overturning since the 2003 Royal Society meeting on abrupt climate change. However, as noted in the previous section, there are many remaining challenges to be addressed.

There are many processes that may contribute to AMOC variability either directly or indirectly. Many of the papers in this volume describe processes that the authors think are critical to AMOC variability. This underlines the need for continuing process studies as well as sustained observations. Additionally, almost all papers underline the need for continued observations of the AMOC to address the outstanding scientific challenges.

The global observation system provided by regular Argo profiles provides a means to study how AMOC changes propagate on regional and basin scales. The long-established meteorological observational network over land can provide evidence on how the AMOC changes may affect local seasonal to interannual climate. The continuing AMOC arrays provide observations of the size and structure of AMOC variability at a range of latitudes. At present, there is a delay of between 6 and 18 months after instruments are recovered from the ocean before an updated AMOC time series can be generated for the arrays. New technology may provide the opportunity to obtain a subset of near real-time observations to produce lower quality but ‘quick-look’ information about the state of the AMOC. In addition, continuing observations of the export of deep waters from the Labrador Sea are needed to document changes in polar and subpolar water mass formation and export.

The RAPID and OSNAP arrays have provided a small number of examples of notable interannual change or events in the AMOC that have advanced our mechanistic understanding. An example is the 2009–2010 event when the AMOC at 26° N decreased by approximately 30% over an 18-month period and then largely recovered. Then after 2014, the subtropical AMOC has been in a reduced state by approximately 2 Sv or approximately 12%. These events were seemingly due to variations in wind forcing, but these AMOC changes have been manifest principally in reduced southward flow of LNADW, with no reduction in UNADW, leading to questions about how wind forcing can impact the deepest layers. Present-day models, both coupled climate and ocean only, do not reflect changes in deep water formation adequately in the Labrador, Irminger and Nordic Seas and improvements are necessary to understand how and why the deep circulation changes.

The subpolar North Atlantic has experienced the largest freshening event in the last 120 years [52]. It is critical to understand where this freshening originates and what effects it has on the AMOC. It is not clear whether present models can simulate such an event. Observing more AMOC ‘events' and careful analysis of their effects and of the post-event behaviour of the overturning will provide more insights. For example, the 2009–2010 event led to a tri-pole SST pattern that is linked to NAO variations, so did it cause the NAO in the following year? Did the reduction in the subtropical AMOC since 2014 contribute to a deepening of the ‘warming hole’ south of Iceland? Has the associated reduction in subtropical heat transport been mirrored in reduced ocean heat loss to the atmosphere north of 26° N? Salinity changes associated with AMOC variations have not been widely investigated, though the reduced subtropical AMOC salt transport since 2014 has been suggested as explanation to the freshening within the subpolar North Atlantic [10]. However, alternative explanations for the subpolar freshening that do not invoke the subtropical AMOC have also been given [52,64]. In models, substantial freshening over the subpolar/polar region leads to shut down of the AMOC [55], and the AMOC arrays are well placed to provide early warning of such a catastrophic tipping point being reached, potentially triggered by Arctic changes.

The value of continuing to observe the AMOC is clear, both from a scientific and a policy perspective (Henderson, meeting presentation), but the carbon and financial costs of doing so over the long-term present a further challenge, especially if those observations are to be made at different latitudes/locations in the Atlantic. There is a clear need to develop lower cost means of observing the AMOC, and there are ongoing studies that are seeking to address this challenge.

Over the next few years, observations will continue to be made using the approaches described during this meeting but the aim is to evolve these into long-term sustained observations at much reduced costs. It remains to be seen whether this will prove achievable. Nevertheless, there will continue to be a need for long-term sustained observations of AMOC and for a new generation of researchers to tackle the scientific challenges that the observations will raise. As the title of the NRC [4] report states there are undoubtedly more ‘inevitable surprises' to come.

**Table:** 

Papers presented at the meeting	
(recordings of the talks are available at: royalsociety.org/science-events-and-lectures/2022/12/atlantic-overturning/)	
*Brown	AMOC and carbon across the Atlantic
Buckley	The role of buoyancy forcing in the Atlantic Overturning Circulation
†Caesar	Evolution of the AMOC: From decades to Millenia
Chafik	Subpolar AMOC between Greenland and Scotland: large-scale mechanisms and linkages
*de Jong	The AMOC and ocean salinity
Frajka-Williams	Should AMOC observations continue - how and why?
Haine	Arctic freshwater impact on the AMOC: status and prospects
*Henderson	Implications of Atlantic circulation on UK policy
Jackson	Challenges simulating the Atlantic meridional overturning circulation in climate models
Johns	Towards two decades of Atlantic Ocean mass and heat transports at 26.5° N
Le Bras	Labrador Sea Water spreading and the Atlantic Meridional Overturning Circulation
Liu	AMOC Modelling for Past Climate and Its Relevance to Present and Future
Lozier	The meridional overturning circulation in the subpolar North Atlantic: new results and new questions
Marotzke	RAPID AMOC observations: from theory to an international standard in circulation monitoring
†McCarthy	The big AMOC question stemming from IPCC WG1
Robson	AMOC, AMV and climate
*these speakers were unable to provide written contributions to this volume	
†these speakers have written a joint paper	
In addition, there were 23 posters presented at the meeting on ongoing research into the AMOC.	

## Data Availability

This article has no additional data.
